# Full Response of a Localized Renal Tumour after Reduced-Intensity Conditioned Hematopoietic Stem Cell Transplantation

**DOI:** 10.1155/2009/879765

**Published:** 2009-11-12

**Authors:** Anne-Claire Gac, Sylvain Chantepie, Michel Leporrier, Oumedaly Reman

**Affiliations:** Service d'Hématologie Clinique, Centre Hospitalo-Universitaire, 14000 Caen, France

## Abstract

Graft versus tumor effect has been described in solid metastatic tumor. We reported here the case of a patient treated for an acute myeloid leukemia with reduced-intensity conditioned allograft and the effect of this procedure on concomitant of renal localised cancer. The effect of immune-mediated cytotoxicity on renal cancer is the more consistent explanation to understand the necrosis of this tumor. Any case of RIC allograft has been reported before to treat localised renal tumor.

## 1. Introduction

The effect of immune-mediated cytotoxicity on renal cancer has been clearly outlined in the last decades by several lines of evidence. We reported here a patient with bone marrow failure treated with RIC allograft and the effect of this procedure on a concomitant localised renal cancer.

## 2. Case Report

A 52-year-old man was referred in 1980 in our department because of severe pancytopenia (platelets: 8 G/L; hemoglobin: 8.3 g/d; MCV: 87 fl, reticulocytes: 0.5% (14000/microL); WBC : 2.1 G/L; Neutrophils: 16%; Lymphocytes: 70%; Monocytes: 4%). Bone marrow was aplastic on histological examination. He was then treated with packed red cells and platelets transfusions and horse antithymocyte globulin with a full response. The disease relapsed in 1997 with the same presentation. After an ineffective course of ciclosporin, a partial response was obtained with a second course of rabbit antithymocyte globulin and high dose androgen therapy. Subsequently, myelodysplasic features appeared with chromosome 7 monosomy, which evolved to a smouldering transformation in AML 6 in April 2004.

**Figure 1:**
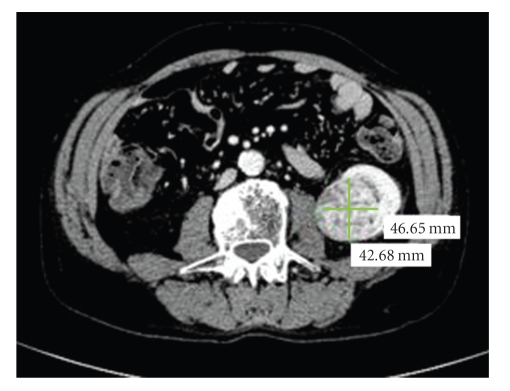
CT scan showing left renal tumor before RIC HSCT.

At the same time, a left kidney tumor (46 × 42 mm) was discovered on computer tomography scan (Figure 1). This renal mass was interpreted as a clear cell carcinoma. However, given the slow progression of the tumor and the poor haematological status, biopsy and nephrectomy was first differed. In June 2005, a reduced-intensity conditioned (RIC) allograft was performed with an unrelated stem cell donor mismatched on DRB3 antigen and on split Cw. The preparative regimen consisted of 30 mg/m^2^ of fludarabine on days -1, -2 and -3 and total body irradiation at 2 grays on day -1. The patient received a dose of 6.11 × 106 CD34/kg.

Prevention of graft versus host disease was based on ciclosporine 3 mg/kg/day started at day 1 and switched on day 6 on mycophenolate mofetil 15 mg/kg twice a day and prednisone 1 mg/kg/day because of an increasing plasma creatinine.

The immediate course was uneventful with a complete full donor-type haematological reconstitution on day 42. At this time, prednisolone was decreased; on day 85, a skin and gut grade II acute graft versus host disease developed which was treated with high dose methylprednisolone and mycophenolate mophetil. At that time, the left kidney tumor measured 50 mm and his aspect remained unchanged on CT scan. Acute GVHD quickly resolved and in May 2006 chronic skin and pulmonary GVHD developed. Subsequent scans showed stability of the renal tumor.

Finally, in September 2006, as the patient has recovered a good clinical and haematological status (haemoglobin: 13.1 g/dL; platelets: 110 G/L; leucocytes: 5.1 G/L with normal differential count), a left nephrectomy was performed. The tumor size was 60 mm × 40 mm. On histological analysis, the tumor process was fully necrosed. Adjacent kidney parenchyma was normal, kidney veins were free of tumor and no metastasis disease was found.

## 3. Discussion

In this case, we could not document precisely the histological type of this kidney cancer, as it was completely necrosed at time of nephrectomy. However, the aspect on the CTscan is favoring the diagnosis of renal cell carcinoma with a strong evidence.

The mechanism accounting for the tumor regression deserves discussion. The tumor necrosis was delayed nine months after the administration of the allograft conditioning regimen.

Spontaneous tumor regression can be hypothesised, but the true incidence of this phenomenon is probably less than 1% and the majority of documented spontaneous regressions involve metastasis spread of renal cell carcinoma, not primitive tumor.

An immune-mediated necrotic process of the kidney cancer is a more consistent explanation in this case. This phenomenon has been clearly illustrated by several lines of evidence in the past. Rosenberg et al. [1] have studied the effects of adoptive immunotherapy with lymphocyteactivated killer (LAK) cells plus interleukin-2 in 157 patients with metastatic cancer in which 36 who had renal-cell cancer. Among 36 evaluable patients treated with LAK cells plus interleukin 2, 12 objective tumor regressions were observed.

Brouwenstijn et al. [2] have isolated and characterized tumor specific CTL from peripheric blood lymphocytes in a patient with RCC and from tumor infiltrating lymphocytes from an other one. This data supports the assumption that common RCC tumor antigens could be recognized by CTL.

Similarly, there is some evidence that bone marrow transplant could reverse the spread of metastatic renal cell carcinoma [3]. Barkholt et al. [4] reviewed 124 patients with metastatic renal cell carcinoma, who received RIC HSCT, have noticed a tumor spread improvement after the procedure in 29% of these cases.

Posttransplant DLI and chronic GVHD improved the patient's survival. TRM was 16%, patients with less than three metastatic locations and a Karnofsky score >70% clearly had a benefit from HSCT. Childs et al. [5] have reported similar results on a groups of 19 patients. On the other hand, Rini et al. [6], reporting on twenty-two patients with advanced renal cell carcinoma treated by HSCT, observe no objective cancer response, despite the occurrence of chronic GVHD. They explained these poor results by a selection of patients with advanced and bulky disease at the time of the grafting, limiting the GVT effect.

A direct cytotoxic effect involving donor lymphocytes issued from the graft is a consistant hypothesis. Harlin et al. [7] have observed in 15 RCC patients treated with RIC allograft, increasing ratio of CD8+ to CD4 T cells, and production of gamma-IFN and IL-2 at the time of clinical responses (generally on day 180 after transplantation). Tykodi et al. [8], monitoring 8 patients, after an RIC HSCT for metastatic renal cell carcinoma, demonstrated the presence of CD8+ CTL able to recognize minor H antigens on tumors cells and hypothesized that these cells could contribute to the GVT effect. On RCC murine models treated with HSCT, Horano et al. [9] have shown that most of the tumor infiltrating lymphocytes were host-derived cells. The level of donorderived lymphocytes gradually decreased over time and was undetectable 120 days after DLI.

In our patient, such a mechanisme remains the more consistent explanation. To our knowledge, a complete necrosis of a localised renal tumor in relation with RIC allograft effect has not been previously reported.
